# Generation of Virtual Non-Contrast CT From Intravenous Enhanced CT in Radiotherapy Using Convolutional Neural Networks

**DOI:** 10.3389/fonc.2020.01715

**Published:** 2020-09-08

**Authors:** Gao Liugang, Xie Kai, Li Chunying, Lu Zhengda, Sui Jianfeng, Lin Tao, Ni Xinye, Dai Jianrong

**Affiliations:** ^1^Radiotherapy Department, Second People's Hospital of Changzhou, Nanjing Medical University, Changzhou, China; ^2^Center for Medical Physics, Nanjing Medical University, Changzhou, China; ^3^School of Biomedical Engineering and Informatics, Nanjing Medical University, Nanjing, China; ^4^Radiotherapy Department, Cancer Hospital Chinese Academy of Medical Sciences, Beijing, China

**Keywords:** convolutional neural networks, radiotherapy, dose, enhanced CT, virtual non-contrast CT

## Abstract

**Objective:** To generate virtual non-contrast (VNC) computed tomography (CT) from intravenous enhanced CT through convolutional neural networks (CNN) and compare calculated dose among enhanced CT, VNC, and real non-contrast scanning.

**Method:** 50 patients who accepted non-contrast and enhanced CT scanning before and after intravenous contrast agent injections were selected, and two sets of CT images were registered. A total of 40 and 10 groups were used as training and test datasets, respectively. The U-Net architecture was applied to learn the relationship between the enhanced and non-contrast CT. VNC images were generated in the test through the trained U-Net. The CT values of non-contrast, enhanced and VNC CT images were compared. The radiotherapy treatment plans for esophageal cancer were designed, and dose calculation was performed. Dose distributions in the three image sets were compared.

**Results:** The mean absolute error of CT values between enhanced and non-contrast CT reached 32.3 ± 2.6 HU, and that between VNC and non-contrast CT totaled 6.7 ± 1.3 HU. The average CT values in enhanced CT of great vessels, heart, lungs, liver, and spinal cord were all significantly higher than those of non-contrast CT (*p* < 0.05), with the differences reaching 97, 83, 42, 40, and 10 HU, respectively. The average CT values of the organs in VNC CT showed no significant differences from those in non-contrast CT. The relative dose differences of the enhanced and non-contrast CT were −1.2, −1.3, −2.1, and −1.5% in the comparison of mean doses of planned target volume, heart, great vessels, and lungs, respectively. The mean dose calculated by VNC CT showed no significant difference from that by non-contrast CT. The average γ passing rate (2%, 2 mm) of VNC CT image was significantly higher than that of enhanced CT image (0.996 vs. 0.973, *p* < 0.05).

**Conclusion:** Designing a treatment plan based on enhanced CT will enlarge the dose calculation uncertainty in radiotherapy. This paper proposed the generation of VNC CT images from enhanced CT images based on U-Net architecture. The dose calculated through VNC CT images was identical with that obtained through real non-contrast CT.

## Introduction

Intravenous iodine contrast-enhanced computed tomography (CT) is usually used in radiotherapy to improve the contrast between tumors and normal tissues, allowing oncologists to accurately delineate the target region and normal tissues (1–3). A high-density contrast medium containing iodine is intravenously injected into the patient before scanning, followed by CT scanning to obtain enhanced CT images. In enhanced CT, specific organs contain considerable contrast medium, giving rise to a remarkable increase in local CT value. This condition improves the contrast ratio of these organs but enlarges the uncertainties in radiotherapy dose calculation (4–6). The CT values and relative electron densities of specific organs in enhanced CT are evidently overestimated in comparison with non-contrast CT; thus, errors occur in radiotherapy dose calculation. Xiao et al. (7) investigated the differences between enhanced and non-contrast CT when applied to dose calculation under three-dimensional conformal radiation therapy (3DCRT), intensity-modulated radiation therapy (IMRT), and stereotactic body radiation therapy (SBRT) and assumed the negligible difference in 3DCRT dose calculation; thus, this method can be directly applied to 3DCRT dose calculation. However, the minimum dose in the planned target volume (PTV) for SBRT and IMRT was overestimated by 2.71%, whereas the maximum dose was underestimated by 1.36%. In the study of Li et al. (8), the difference between the average CT values for the heart in enhanced and non-contrast CT was 136.4 HU; the γ passing rate was between 96.54 and 99.99% under 3% absolute dose difference/3 mm distance-to-agreement criteria for lung cancer patients; thus, enhanced CT can give rise to minimal dose calculation difference for lung cancer patients. Hwang et al. (9) investigated the influence of enhanced CT on dose calculation of proton beam radiotherapy and noted that the distal range error of proton beam caused by enhanced CT reached as high as 1 cm. Thus, the CT values of heart and great vessels must be corrected to apply enhanced CT to proton beam radiotherapy.

The direct use of enhanced CT in radiation dose calculations can lead to errors, which are usually avoided in two ways. In the first method, rigid registration of enhanced and non-contrast CT images is conducted for the patient, and PTV is delineated in the enhanced CT image and mapped to the non-contrast CT image. Next, dose calculation is performed on the non-contrast CT image. This method requires the patient to accept radiation of CT scanning twice. Moreover, the registration of the two images will generate additional errors, thereby enlarging the radiotherapy uncertainty. In the second method, the enhanced region influenced by contrast medium is manually delineated and overrides a certain electron density, and dose calculation is directly performed on the enhanced CT image. This method is time consuming and seriously affected by human experience. The deep learning technology based on convolutional neural networks (CNN) has been extensively applied to medical image processing (10–15). Researchers have achieved ideal results using deep learning in various fields, such as image segmentation (16, 17), CT image denoising and artifact reduction (18), image registration (19, 20), and radiotherapy response prediction (21). Zhang and Yu (22) introduced CNN to obtain prior image and desirable results in metal artifact reduction in CT. For magnetic resonance (MR)-guided radiotherapy, Fu et al. (23) used 2D and 3D CNNs to generate pseudo-CT image from the MR image of the T1 phase; Gupta et al. (24) used U-Net neural network to generate a pseudo-CT image from a MR image with sagittal view and calculated the dose distribution based on pseudo- and real CT images. However, the use of deep learning method in image transformation from enhanced CT to non-contrast CT has not been studied.

In this study, a method of generating VNC CT image from enhanced CT image through U-Net (25) was proposed. Dose distribution was calculated based on non-contrast, enhanced, and VNC CT images, and their differences were compared.

## Materials and Methods

CT images of several patients who underwent non-contrast and enhanced CT scanning of the chest were selected. The CT images were scanned on Siemens CT (SOMATOM Force, Germany). The scanning parameters were as follows: tube voltage, 110 kVP; tube current, 400 mA; layer thickness, 3 mm; scanning spatial resolution, 0.72 × 0.72 mm^2^ to 0.97 × 0.97 mm^2^; size of reconstructed image, 512 × 512. The patients first received non-contrast CT scanning while retaining their body positions. The nurse performed intravenous contrast agent injections using a high-pressure pump for enhanced CT scanning in venous phase. The time interval between enhanced and non-contrast CT scanning was <3 min. The patients held their breath after inspiration in the non-contrast and enhanced scanning process. Thus, the deformation difference in the two images caused by breathing movement was as minimal as possible.

Non-contrast CT images from each group of CT images were captured as fixed images, whereas enhanced CT images were used as moving images to conduct 3D rigid registration of CT image through 3D affine transformation. After registration, the images were reviewed by a senior radiotherapy oncologist who observed location differences among organ tissues in the non-contrast and registered enhanced CT images and excluded images with considerable deformation errors. Consequently, the CT images of 50 patients were selected, and each group of images included 60-slice non-contrast and 60-slice enhanced CT images.

**Figure 1:**
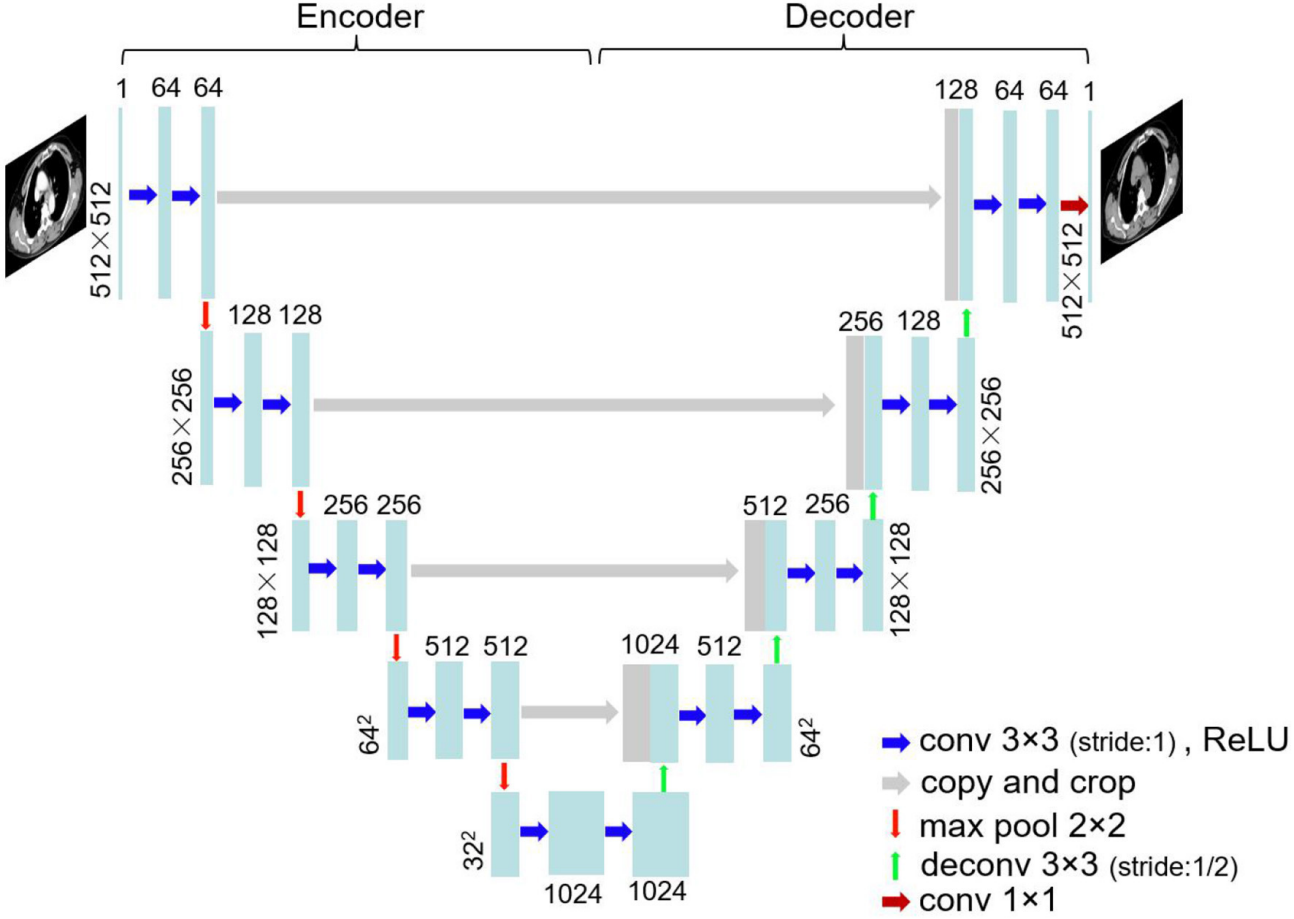
U-Net architecture.

The images were trained using U-Net architecture. U-Net (25), which was proposed in biomedical image segmentation task for the first time, has been extensively used by virtue of small training data requirements and good effect. Figure 1 shows the network structure. U-Net is a left–right symmetrical neural network structure containing encoder and decoder parts. Four downsampling processes were used to extract image features in the encoder part, and the decoder part contains four upsampling processes that recover the feature map to the original image resolution. Convolution kernels (3 × 3) were used at a stride of one. Zero padding was conducted before the convolution. Thus, the image size was unchanged before and after convolution. The convolution layer was followed by batch normalization and ReLU activation. Maximum pooling with a 2 × 2 window and a stride of two was carried out in the encoder part, whereas a deconvolution at stride of 1/2 was conducted in the decoder part. The enhanced CT image was considered the input and the corresponding non-contrast CT image the output. The CT images of 40 patients, including 2,400-slice non-contrast and 2,400-slice enhanced CT images, were obtained as the training data. Data augmentation was implemented by random rotation of image and also by randomly cropping a section of each image for training. The remaining images of 10 patients were used as the test data. The sum of mean absolute error (MAE) and mean square error (MSE) served as loss function:

$$\begin{array}{l} MAE\left(X,\;Y\right)=\frac{1}{n}\overset{n}{\underset{i=1}{\sum }}|X_{i}-Y_{i}|\\ MSE\left(X,\;Y\right)=\frac{1}{n}\overset{n}{\underset{i=1}{\sum }}{\left(X_{i}-Y_{i}\right)}^{2}\\ L\left(X,\;Y\right)=MAE\left(X,\;Y\right)+MSE\left(X,\;Y\right) \end{array}$$

where X and Y are the compared CT images, and *X*_*i*_ represents the CT value of the i(th) pixel in CT image. Minibatch gradient descent optimization algorithm was adopted in the training with a batch size set to 12.

### Treatment Planning

The test data included ten esophagus cancer patients who received radiotherapy. The non-contrast and enhanced CT images of the ten patients and their VNC CT images, which were generated through U-Net, were imported into the commercial treatment planning system (Monaco 5.11, Elekta, Sweden). Senior oncologists delineated the PTV and important protective organs, including the heart, great vessels, lungs, liver, and spinal cord on the non-contrast CT images. The delineated PTV and protective organs were replicated in enhanced and VNC CT images to compare the differences in the organs in terms of the average CT values on the three types of CT images.

**Figure 2:**
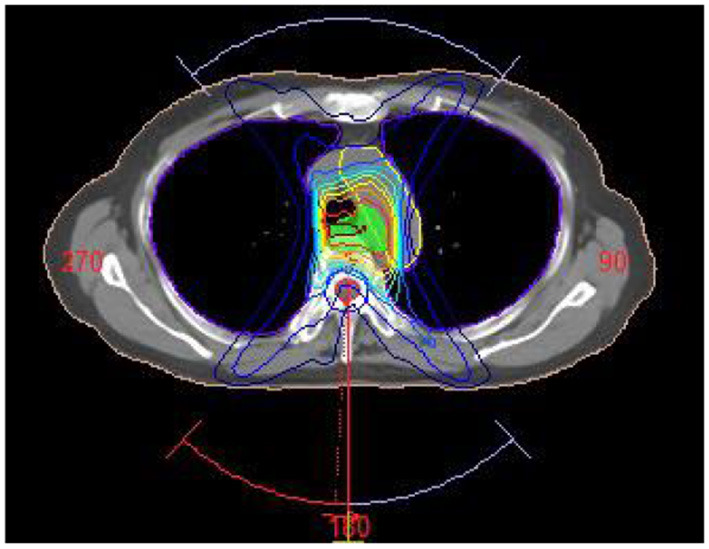
Arc irradiation field in radiotherapy plan.

A treatment planning study was performed in Monaco 5.11. For each patient in the treatment planning system, an irradiation plan was designed with Elekta Infinity accelerator whose multileaf collimator consists of 80 pairs of leaves (width: 0.5 cm). Volumetric modulated arc radiotherapy plan was designed, and each PTV was given at the prescribed dose of 60 Gy/30 fractions. Three irradiation fields were designed in planning (gantry angle from 181 to 220°, 320 to 40° and 140 to 180°) to avoid the lung area as shown in Figure 2. Each field contained three arcs, and the maximum number of control points on each arc was set at 200. Monte Carlo algorithm was used in dose calculation, computational grid size was set at 3 mm, and calculation uncertainty was 2% at each control point. The radiotherapy plan was designed and optimized on the non-contrast CT images. During the optimization, 95% of PTV was covered by prescribed dose (60 Gy), the percent volume of lung covered by 20 Gy was <30%, and the maximum dose of spinal cord was <45 Gy. With these primary constraints satisfied, the average dose of protective organs were set as low as possible. After optimization, the dose distribution was calculated in the non-contrast CT images. The irradiation field was copied to enhanced CT and VNC images, and dose was directly calculated without plan optimization. The dose distribution differences in the three CT images were compared. Wilcoxon signed-rank test was adopted for comparison of the average CT and mean dose values.

## Results

**Figure 3:**
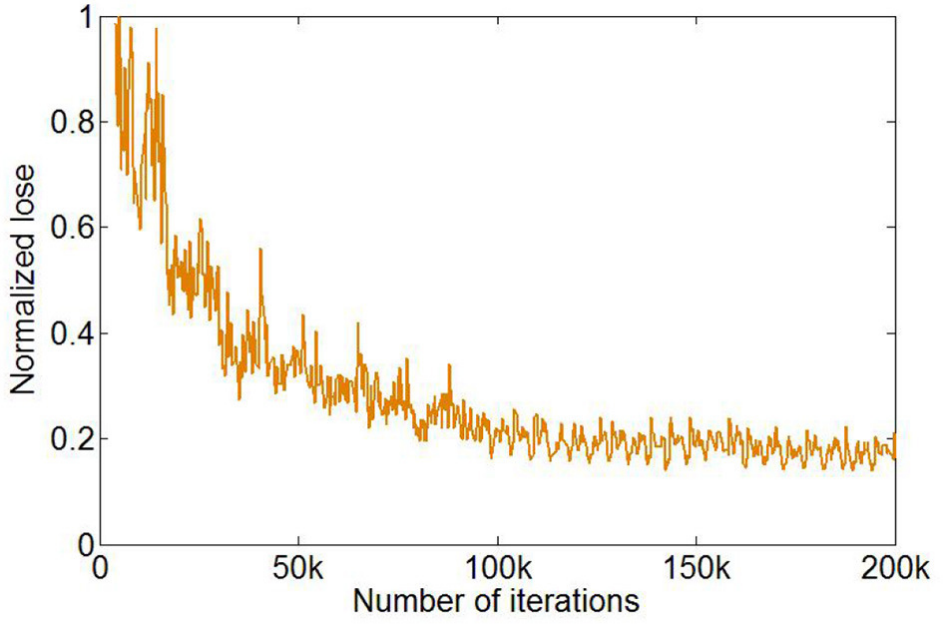
Changes in loss function value during training with the number of iterations.

The training of U-Net was stopped after 1,000 epochs, and the total number of iterations was 200,000. The loss function in the training process reached a plateau (Figure 3).

**Figure 4:**
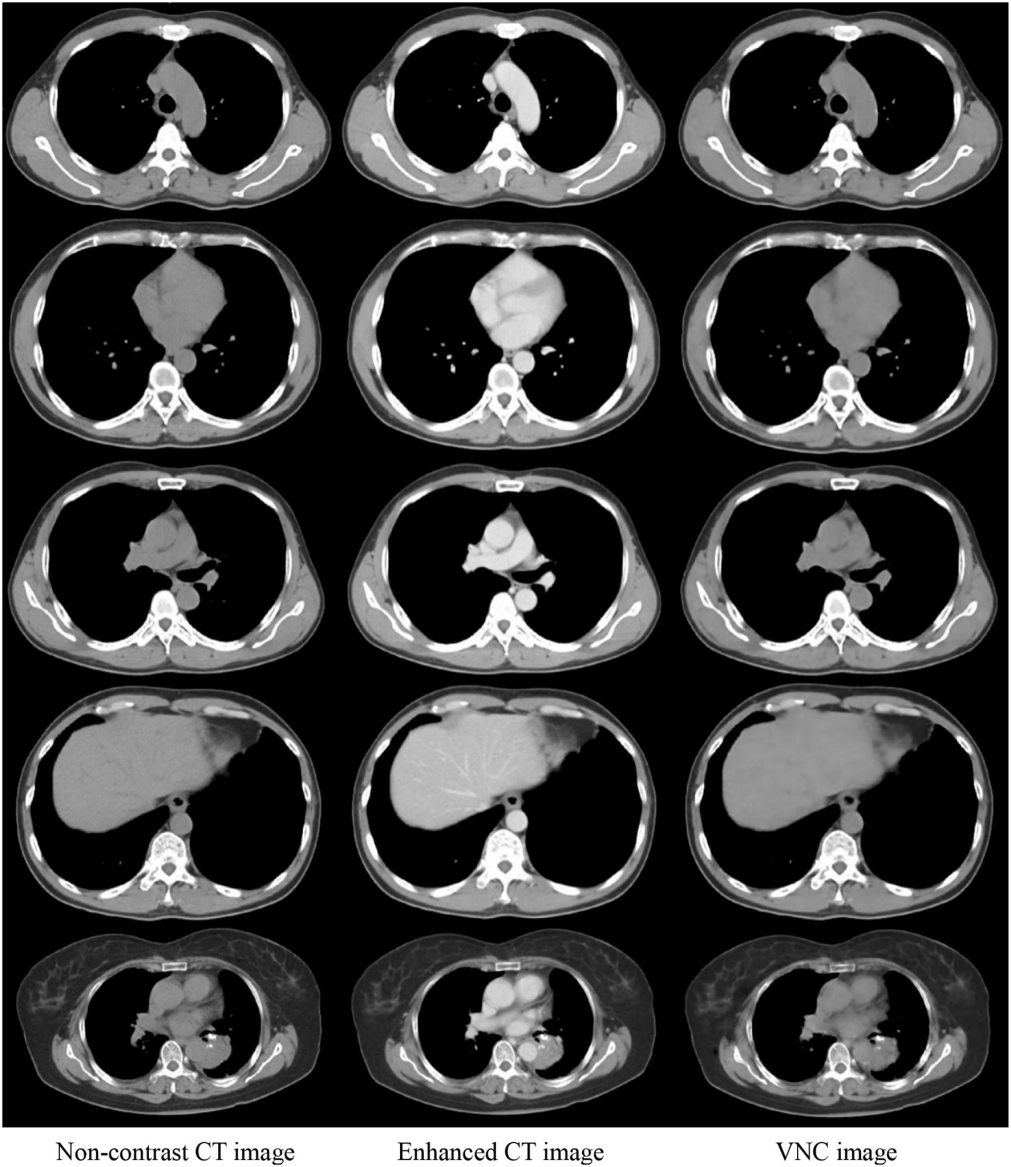
Comparison of three CT images in the same slice. Real non-contrast CT, enhanced CT, and VNC images generated through U-Net at the left, middle, and right columns, respectively.

The enhanced CT images in the test data were inputted into the trained U-Net to generate VNC CT images. Figure 4 shows the real non-contrast, enhanced, and VNC CT images at the left, middle, and right columns, respectively. The brightness values of enhanced CT images in great vessels, heart, and liver were all notably higher than those of non-contrast CT images. However, the VNC CT images were similar to non-contrast CT images. The window level and width in the CT images reached 40 and 400 HU, respectively.

**Figure 5:**
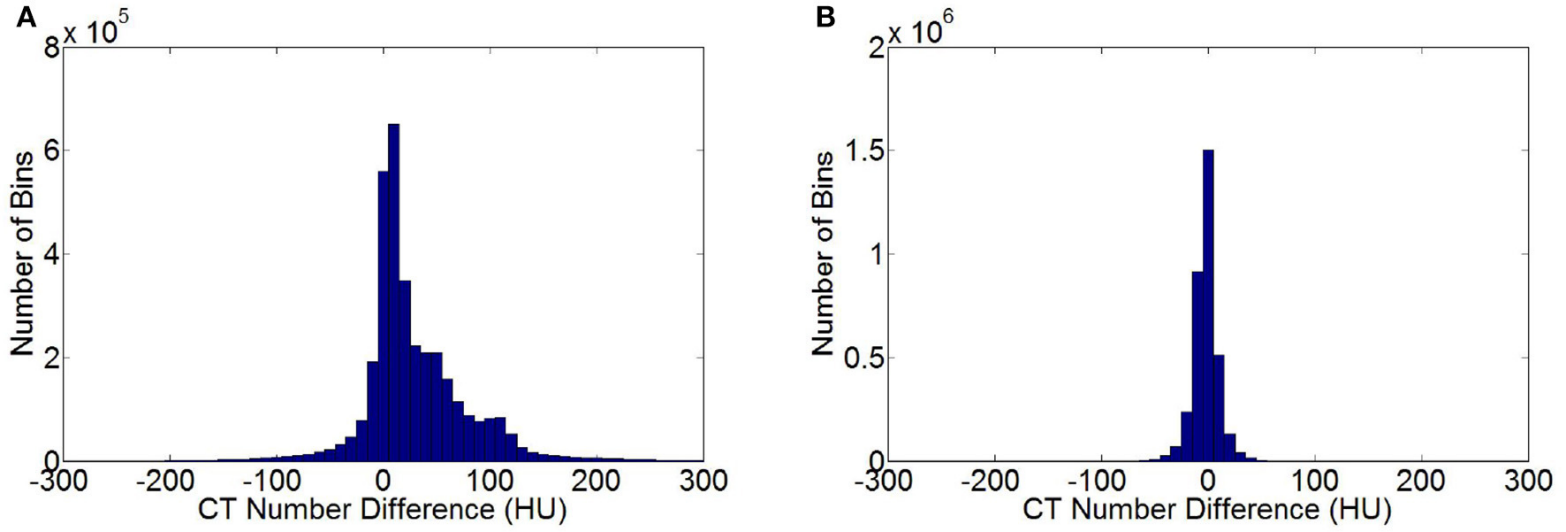
Statistical histogram of CT value differences. **(A)** Histogram of CT value differences between enhanced and non-contrast CT images. **(B)** Histogram of CT value differences between VNC and non-contrast CT image.

Figure 5A shows the statistical histogram of CT value difference between the enhanced and non-contrast CT images of a patient. The difference in the value image was obtained by deducting the CT value of a non-contrast image from that of an enhanced image. The number of pixels in the difference value image in each interval of CT value was calculated with 10 HU as a unit. A histogram concentrating on 0 HU, indicates a high similarity between two images. If the two images were exactly the same, then all pixels were distributed within the unit corresponding to 0 HU. Most of the CT value differences between the enhanced and non-contrast images were distributed within a positive interval, and numerous pixels were distributed at 100 HU or higher. Several organs adsorbed the contrast medium in the enhanced image. Thus, the CT value was notably larger than that in the non-contrast image. Figure 5B shows the statistical histogram of CT value differences between VNC and non-contrast CT images. Pixels were under the concentration distribution of 0 HU in the histogram. Several pixels were >30 HU, and the CT value difference between VNC and non-contrast CT images was minimal. The MAE of enhanced and VNC CT images in HU values in the test data was calculated, excluding the *in-vitro* air region, with a non-contrast CT image as reference image. The MAE of enhanced and VNC CT image reached 32.3 ± 2.6 and 6.7 ± 1.3 HU, respectively.

Table 1 lists the average CT values for several organs in enhanced CT, VNC, and non-contrast CT images. A senior radiotherapy oncologist delineated the organs, such as heart, great vessels, lungs, liver, and spinal cord, in the non-contrast, enhanced, and VNC CT images of ten patients and calculated the average CT values of the organs. The CT values of the heart, great vessels, lungs, liver, and spinal cord in the enhanced CT images were all significantly greater than those in the non-contrast CT images (*p* < 0.05), where the average CT value differences of great vessels reached the maximum of up to 97 HU. The average CT value difference of the heart reached 83 HU, whereas those of lungs, liver, and spinal cord totaled 42, 40, and 10 HU, respectively. VNC and real non-contrast CT images showed no significant difference in the HU value. The difference in average CT values of the lungs between the two images was 4 HU, whereas those of heart, great vessels, liver, and spinal cord were all <2 HU.

**Table 1:** Average CT values of different organs in the three types of CT images.

	**Average CT values of organs (HU)**				
	**Heart**	**Great vessels**	**Lungs**	**Liver**	**Spinal cord**
Non-contrast CT	26.5 ± 5.2	42.6 ± 2.3	−698.6 ± 65.8	52.4 ± 4.6	31.2 ± 2.3
Enhanced CT	109.2 ± 16.2	139.3 ± 14.8	−656.4 ± 76.3	92.2 ± 8.7	41.1 ± 3.7
Virtual non-contrast (VNC) image	25.2 ± 6.8	41.3 ± 3.9	−702.4 ± 72.7	52.8 ± 6.5	32.6 ± 2.7

**Figure 6:**
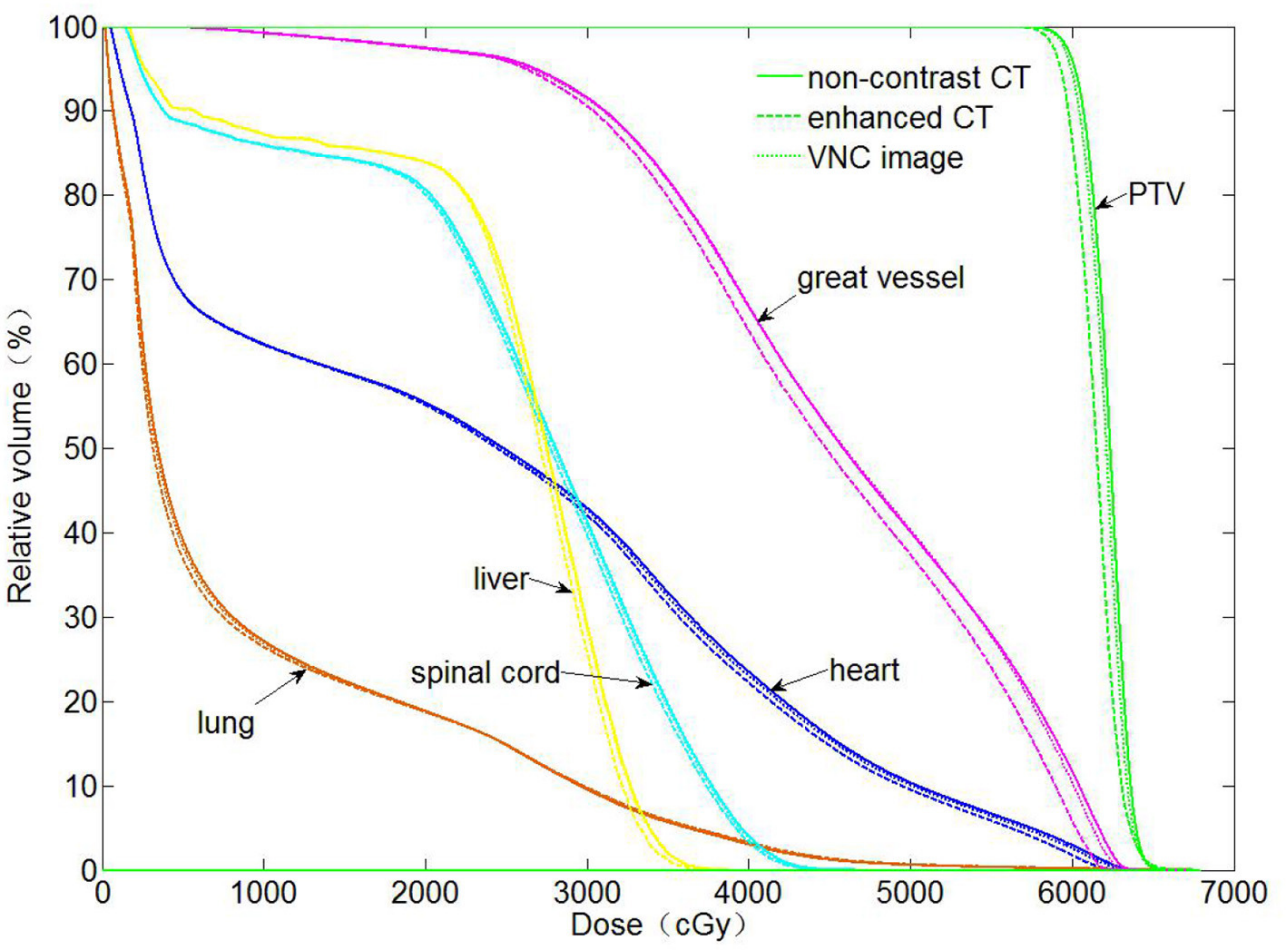
Dose volume histogram for the organs calculated using the three types of CT images.

**Figure 7:**
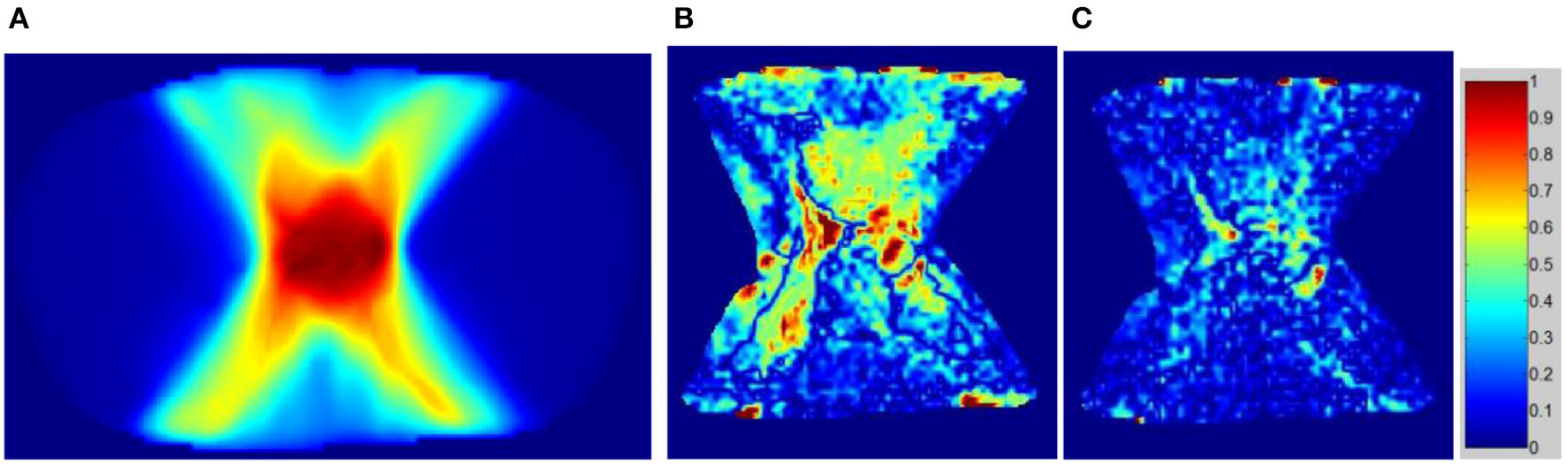
γ passing rate distribution on enhanced and VNC CT images with the dose of non-contrast CT image used as the criterion. **(A)** Dose distribution on the non-contrast CT image, **(B)** γ distribution of the enhanced CT image, and **(C)** γ distribution of the VNC CT image.

The dose distributions of PTV and organs in the three types of CT images were calculated. Non-contrast CT dose was used as the reference to compare the relative dose difference in enhanced and VNC CT images. The three types of images exhibited no statistical difference in the comparison with the maximum and minimum doses of PTV and maximum dose of spinal cord. Enhanced and non-contrast CT presented statistical differences in the mean doses of PTV, heart, great vessels, and lungs (*p* < 0.05). Table 2 shows that the mean doses of enhanced CT for these organs were all remarkably lower than those in non-contrast CT image, where the relative dose difference of great vessels was the maximum, reaching as high as 2.1%. The values for the other organs were all between 1 and 2%. VNC and non-contrast CT images exhibited no statistically significant difference in the mean dose. Figure 6 shows the dose volume histogram of the three types of CT images for the organs of a typical patient. The dose lines for organs in the VNC and non-contrast CT images are superimposed. A remarkable difference can be observed between the dose lines of the enhanced and non-contrast CT image. This difference was especially evident in PTV (green) and great vessels (pink). Considering the dose distribution calculated for non-contrast CT image as reference, the γ passing rates of dose in enhanced and VNC CT images were calculated under 2% absolute dose difference/2 mm distance-to-agreement criteria. The average γ passing rate of the VNC CT images was significantly higher than that of the enhanced CT image (0.996 vs. 0.973, *p* < 0.05). Figure 7 shows a typical cross-sectional γ distribution. The γ distribution obtained for the enhanced CT image showed that considerable dose differences existed around the high-dose region from the non-contrast CT image, but the dose difference from the VNC CT image was negligible.

**Table 2:** Relative dose differences in enhanced and VNC CT images compared with that in non-contrast CT image.

	**Relative dose difference from non-contrast CT (%)**			
	**Mean dose of planned target volume**	**Mean dose of heart**	**Mean dose of great vessels**	**Mean dose of lungs**
Enhanced CT	−1.2 ± 0.5	−1.3 ± 0.4	−2.1 ± 0.7	−1.5 ± 0.4
VNC image	0.1 ± 0.2	−0.1 ± 0.2	−0.2 ± 0.1	−0.2 ± 0.1

## Discussion

The CT values of certain organs were higher than those in non-contrast CT image due to the influence of contrast medium in enhanced CT image, thereby causing inaccurate dose calculation. Although Choi et al. (4) deemed that the dose difference caused by enhanced CT was smaller than 1% when applied to dose calculation in head and neck tumor IMRT, the dose differences in parotid gland and spinal cord showed no significance. Thus, this difference caused no influence on clinical dose evaluation. However, this phenomenon is only restricted to specific body parts of tumor patients. High-precision radiotherapy techniques may amplify the effect of enhanced CT on radiation dose calculation. Xiao et al. (7) pointed out in their study that the influence of enhanced CT on IMRT and SBRT dose calculation was significantly greater than that on 3DCRT. Shin et al. (26) observed that in proton beam radiotherapy dose calculation, the deviation of calculated distal range in the contrast medium from measured range in water reached as high as 3.65 cm in enhanced CT, and 1 cm distal range deviation was produced in the patient plan. The influence of contrast medium must be corrected when enhanced CT is applied to radiotherapy dose calculation in high-precision radiotherapy-like proton radiotherapy and SBRT.

In this paper, VNC CT images were generated from enhanced CT images in the venous phase using U-Net, and the CT value and dose distribution differences among real non-contrast, enhanced, and VNC CT images were compared. In our study, changes in the CT value of heart and great vessels caused by enhanced CT reached the maximum, which agrees with the study result of Hwang et al. (9). The CT values of organs in generated VNC CT images showed no significant differences from those in real non-contrast CT images. The dose calculation in enhanced CT caused significant deviations in the mean doses of heart, great vessels, lungs, and PTV, with the maximum mean dose deviation reaching as high as 2.1% for the great vessels and 1.2% for PTV; these values were greater than those obtained by Xiao et al. (7) and Li et al. (8) (all were <1%) and similar to the result on abdomen, as indicated in the research of Shibamoto et al. (5). These findings are related to tumor location, layout of irradiation field, and dose calculation accuracy. Esophagus cancer radiotherapy plan was adopted in this study, and the irradiation field mostly passed through the heart and great vessels, thereby causing substantial dose differences. In addition, dose distribution was calculated through Monte Carlo algorithm, and calculation uncertainty was set at 2% at each control point to improve the dose calculation accuracy. A small difference was observed between the doses calculated for the VNC and real non-contrast CT images. The mean doses for the organs and PTV exhibited no remarkable differences.

Although the VNC images generated in this study reduced the difference between enhanced and actual non-contrast CT images to a substantial degree, their MAE with real non-contrast CT images (air part *in-vitro* not included) was still 6.7 HU, which might be caused by various factors. The CT images of 40 groups of patients were used to train the network in this study. Increasing the training data may improve the accuracy of image conversion. The original U-Net architecture, which is mainly applied to image segmentation, was used for image generation in this study; thus, the precision was limited to a certain degree. The training accuracy may be improved using specific U-Net-based improved networks (27–29) or generative adversarial network (30). In addition, this part of MAE might be derived from deformation differences existing between enhanced and non-contrast CT images after registration. Although the time interval between the two types of CT scanning (<3 min) was controlled in this study, the patients held their breadth in scanning, and the senior oncologists excluded images with large deformation differences. However, the deformation error caused by organ movement was unavoidable. On the one hand, deformation difference caused errors in network training, thus resulting in the insufficient accuracy of the training network. On the other hand, the deformation difference was transferred from enhanced CT to VNC image in the test process, and several organ contours between VNC and real non-contrast CT images showed no complete overlap, which might have resulted in increased MAE.

## Conclusion

The CT values of organs, including great vessels, heart, and liver, in enhanced CT images were remarkably greater than those in non-contrast CT images. Dose calculation based on enhanced CT will reduce the accuracy in radiotherapy. The VNC images generated from enhanced CT through CNN approximated their real non-contrast CT counterparts. The dose distribution can be accurately calculated based on the VNC images.

## Data Availability Statement

The datasets generated for this study are available on request to the corresponding author.

## Ethics Statement

This study was approved by the Research Ethics Board of the Second People's Hospital of Changzhou, Nanjing Medical University. Written informed consent to participate in this study was not required in accordance with national and institutional guidelines.

## Author Contributions

GL and XK contributed equally to this work, participated in the design of the study, carried out the study, performed the statistical analysis, and drafted the manuscript. LC, LZ, SJ, and LT helped to carried out the study. DJ reviewed and edited the manuscript. NX conceived and designed the study, edited, and reviewed the manuscript. All authors read and approved the final manuscript.

## Conflict of Interest

The authors declare that the research was conducted in the absence of any commercial or financial relationships that could be construed as a potential conflict of interest.
